# Virus-specific host responses and gene signatures following infection with major SARS-CoV-2 variants of concern: role of ZBP1 in viral clearance and lung inflammation

**DOI:** 10.3389/fimmu.2025.1557535

**Published:** 2025-05-09

**Authors:** Amany Elsharkawy, Hamid Reza Jahantigh, Anchala Guglani, Shannon Stone, Komal Arora, Mukesh Kumar

**Affiliations:** ^1^ Department of Biology, College of Arts and Sciences, Georgia State University, Atlanta, GA, United States; ^2^ Center of Diagnostics and Therapeutics, Georgia State University, Atlanta, GA, United States

**Keywords:** SARS-CoV-2, variants of concern, inflammation, pyroptosis, necroptosis, Z-DNA binding protein 1

## Abstract

SARS-CoV-2 can cause severe lung damage due to uncontrolled viral replication or/and excessive inflammation. New variants of concern (VOCs) have raised additional concerns due to disparate pathogenicity and possible enhanced virulence. Herein, using RNA sequencing, we performed a comparative transcriptomic analysis following infection with major VOCs. We evaluated the transcriptional changes induced in the lungs of K18-hACE2 mice following infection with the ancestral B.1 lineage (Wuhan), B.1.1.7 (Alpha), B.1.351 (Beta), B.1.617.2 (Delta), B.1.1.529 (Omicron) variants or mouse-adapted SARS-CoV-2 (MA10). Our work reveals the molecular basis of pathological hallmarks in the lungs associated with SARS-CoV-2 infection. We report that infection with B.1, pre-Omicron VOCs, and MA10 induce similar molecular fingerprints of excessive lung inflammation and immune activation in K18-hACE2 mice. Analysis of differentially expressed genes revealed both shared and variant-specific responses, with key immune markers such as Cxcl10, Zbp1, Ifit3, Isg15, Rsad2, and Irf7 consistently upregulated across variants. Clustering of highly variable genes across samples revealed two variant groups distinguished by upregulation of antigen presentation and immune-related genes (*e.g. Retnla, Saa3, Plac8, Ly6c2, H2-D1, and H2-K1*). Delta, Beta, Alpha, and MA10 showed elevated expression, whereas Wuhan and Omicron exhibited attenuated responses. In addition, we show that Z-DNA-binding protein 1 (ZBP1) plays a role in viral clearance in the lungs after SARS-CoV-2 infection. ZBP1 deficiency resulted in reduced expression of cell death-associated markers and virus-induced cell death in the lungs following MA10 infection. Furthermore, the knockout of ZBP1 resulted in an attenuated inflammatory response with reduced production of proinflammatory cytokines and chemokines and decreased macrophage infiltration in the lungs. These results suggest that ZBP1 plays a role in viral clearance and in enhancing the inflammatory response and virus-induced cell death during SARS-CoV-2 infection. Altogether, our study provides insights into the pathogenesis of SARS-CoV-2 infection in mice, facilitating the identification of biomarkers and the development of potential therapeutic targets.

## 1 Introduction

The COVID-19 pandemic caused by the severe acute respiratory syndrome coronavirus 2 (SARS-CoV-2) is the most significant global public health challenge of the 21st century, causing substantial morbidity and mortality. SARS-CoV-2 is a positive-sense, single-stranded RNA virus that belongs to the Coronaviridae family and the Betacoronavirus genus (1–3). SARS-CoV-2 enters the host cell through receptor mediated endocytosis when the receptor binding domain (RBD) region of the S1 subunit in the spike protein interacts with the angiotensin-converting enzyme 2 (hACE-2) receptor in host cells (4). Since the emergence of SARS-CoV-2 in late 2019, several variants of concern (VOCs) including the B.1.1.7 variant (Alpha), the B.1.351 variant (Beta), the B.1.617.2 variant (Delta), and the B.1.1.529 variant (Omicron), have spurred recurring global infection waves. It has been shown that VOCs confer notable mutations and have the potential for enhanced transmission rates, disease severity, virulence, or immune escape, reducing current treatment and vaccine efficacy (5–10). The first SARS-CoV-2 VOC to emerge was the Alpha variant in the United Kingdom; the Beta lineage was discovered in South Africa, and the Delta variant was first detected in India (11–14). The Alpha variant has a higher transmission rate than the original strain B.1 and has been associated with increased disease severity and mortality. The Alpha variant contains 17 nonsynonymous mutations including mutations in the RBD region such as E484K, S494P, P681H and N501Y (15, 16). Like the Alpha variant, the Beta variant has multiple mutations in the spike protein, including the E484K and D80A mutation, reducing the efficacy of monoclonal antibodies (17, 18). The Delta variant has multiple mutations in the spike protein, including the L452R mutation, which is associated with increased transmissibility and immune evasion (19). Notably, the Omicron variant is the most mutated SARS-CoV-2 variant with a significant number of mutations. The Omicron variant has about 37 mutations within its spike protein, 15 of which in the RBD, contributing to increased transmissibility and immune evasion. Serum samples from COVID-19 convalescent patients or vaccinees had significantly diminished neutralization activity against the Omicron variant (20). Mouse-adapted SARS-CoV-2 variant (MA10) binds to the mouse ACE-2 receptor and results in productive virus replication in the respiratory airway of the wild-type mice (21, 22).

Z-conformation nucleic acid binding protein 1 (ZBP1) is a cytosolic innate immunity sensor. ZBP1 N terminal Z-DNA binding domain (ZBD) binds to Z-DNA, a left-handed double-strand DNA helix (23). Additionally, ZBP1 was reported as an innate sensor to single-stranded negative sense RNA viruses such as Influenza A virus (IAV) (24). ZBP1 has been shown to induce necroptosis mediated by RIPK3 and the downstream effector MLKL, as well as to drive apoptosis in response to viral infections. Furthermore, ZBP1 induces type-I interferon (IFN-I) response and NF-κB signaling, constituting an important line of defense against viral infections (25). Recent studies have reported ZBP1-mediated cell death through the induction of PANoptosis which is a crossed inflammatory cell death that has key features of pyroptosis, apoptosis, and necroptosis (26). We previously reported that West Nile virus (WNV) infection induced a significant increase in ZBP1 expression in mouse brains and in infected primary mouse cells (27). We further showed that ZBP1 deficiency resulted in significantly higher morbidity and mortality following infection with WNV NY99 and WNV Eg101 strains in mice. These reports suggested the role of ZBP1 in restricting the pathogenesis of WNV in mice (28).

In the current study, we compared the transcriptional response in the lungs following infection with Wuhan, major VOCs including Alpha, Beta, Delta and Omicron, and MA10. We further investigated of the role of ZBP1 in SARS-CoV-2 pathogenesis in the lungs. We showed that ZBP1 plays a role in viral clearance and in virus-induced cell death in the lungs following infection with MA10. Additionally, we assessed lung pathology and inflammation during SARS-CoV-2 infection in the absence of ZBP1.

## 2 Materials and methods

### 2.1 *In vivo* mouse challenge experiments

*In vivo* mouse experiments involving infectious SARS-CoV-2 were performed in Animal Biosafety Level 3 laboratory and strictly followed the approved standard operation procedures. The protocol was approved by the Georgia State University Institutional Animal Care and Use Committee (Protocol number A24003). Hemizygous K18-hACE2 mice (2B6.Cg-Tg (K18-ACE2)2Prlmn/J) were obtained from the Jackson Laboratory. K18-hACE2 mice (6–8 mice per group) were inoculated intranasally with the 10^4^ plaque-forming units (PFU) of following SARS-CoV-2 strains, B.1 virus (Wuhan; BEI# NR-52281), B.1.1.7 virus (Alpha; BEI# NR-54000), B.1.351 virus (Beta; BEI# NR- 54008), B.1.617.2 virus (Delta; Northwestern Reference laboratory, Clinical isolate #2333067), B.1.1.529 virus (Omicron; BEI# NR-56461), and mouse-adapted virus (MA10; BEI# NR-55329). On days 3 and 6 post infection, infected K18-hACE2 mice (3–4 mice per group for each time point) were anesthetized using isoflurane and perfused with cold 1X PBS. Collected lung samples were flash-frozen in 2-methylbutane (Sigma, St. Louis, MO, USA) for further analysis (1, 2). Wild-type (WT) C57BL/6J mice were purchased from the Jackson Laboratory (Bar Harbor, ME). ZBP1 knockout (ZBP1^−/−^) mice (nbio155) were obtained from the JCRB Laboratory Animal Resource Bank of the National Institutes of Biomedical Innovation, Health and Nutrition (Osaka, Japan) (28). For body weight measurement, WT and ZBP1^−/−^ mice (n=7 per group) were inoculated with 10^4^ plaque-forming units (PFU) of MA10 virus. In independent experiments, MA10-infected WT (n=10) and ZBP1^−/−^ (n=10) mice were euthanized at days 3 and 6 post infection (n=5 for each time point). An equal number of male and female mice were used for each group.

### 2.2 RNA extraction and reverse transcription quantitative PCR

After tissue homogenization and lysis in RLT buffer (Qiagen, Catalog# 79216), total RNA was extracted from the lungs using a Qiagen RNeasy Mini kit (Qiagen, Catalog# 74104) following the manufacturer’s instructions. To measure the purity and quantity of total RNA, NanoDrop spectrophotometer (Thermo Fisher Scientific, Waltham, MA, USA) was used. Next, cDNA was synthesized from 1000 ng of RNA using an iScriptTM cDNA synthesis kit (Biorad, Catalog# 1708891). The cDNA was diluted with RNAse-free water, and 2 µL of cDNA was used for qPCR using SsoAdvanced™ Universal SYBR^®^ Green Supermix (Biorad, Catalog# 1725271). Fold change of genes for infected tissue was calculated against control samples after normalizing to the *Gapdh* gene (**Table 1**). Viral RNA levels were measured with primers and probes specific for the SARS-CoV-2 N gene (Integrated DNA Technologies, Catalog# 10006713). Viral genome copies were calculated using a standard curve and expressed per μg of total RNA (29, 30).

**Table 1:** Primer sequences used for RT-qPCR.

Gene (Accession No.)	Forward Primer Sequence (5’->3’)	Reverse Primer Sequence (5’->3’)
*Casp3* (NM_00128440)	GCTTGGAACGGTACGCTAAG	AGCCTCCACCGGTATCTTCT
*Casp7* (NM_007611)	CGTGGGAACGATGACCGA	GGATTCATCAACGAACGGCG
*Casp6* (NM_009811)	AAGTGTTCGATCCAGCCGAG	CAGGTTGTCTCTGTCTGCGT
*Casp9* (NM_015733)	CCGTGGGAGCTGTTTTGATT	TCCTGCCTGCTGAATATCCTC
*Casp4* (NM_007609)	CCTGCAGAGGTGGGAACTC	AAGGTTGCCCGATCAATGGT
*Il-18* (NM_008360)	GGCTGCCATGTCAGAAGACT	ACACAAACCCTCCCCACCTA
*Gsdmd* (NM_026960)	ATCAAGGAGGTAAGCGGCAG	CCTTCTCCCATGCCTGACAA
*Tnf-α* (NM_013693)	CCAGTCTGTATCCTTCTAA	TCTTGTGTTTCTGAGTAGT
*Cxcl10* (NM_021274)	GGTCTGAGTCCTCGCTCAAG	GTCGCACCTCCACATAGCTT
*Zbp1* (NM_001139519)	GGCAGAAGCTCCTGTTGACT	CTGTCCTCCTTCTTCAGGCG

### 2.3 RNA-seq and data analysis using ingenuity pathway analysis

On days 3 and 6 after inoculation, three to four SARS-CoV-2-infected mice from each infection group were euthanized using isoflurane and perfused with 1XPBS. Lung tissue samples from SARS-CoV-2-infected mice and mock-infected controls were collected and flash frozen in 2-methylbutane. RNA was extracted from tissue samples as described above. Samples with an RNA integrity number (RIN) greater than 7.0 on the 2100 Bioanalyzer were used. TruSeq Stranded mRNA Sample Preparation Guide and TruSeq Stranded mRNA LT Sample Prep Kit was used to construct the library. Whole transcriptome sequencing was performed for each SARS-CoV-2-infected mouse sample and mock-infected controls using Illumina NovaSeq 6000. The quality of produced data was determined by the Phred quality score at each cycle; reads over a Phred quality score 20 were accepted as good quality. Next, the Trimmomatic program v0.38 was used to remove adapter sequences and low-quality bases lower than three from the ends (31). Reads with length shorter than 36bp were also dropped to produce trimmed data. Trimmed reads were then mapped to the UCSC mm10 reference genome with HISAT2 v2.1.0 (46). After the read mapping, StringTie v2.1.3b was used for transcript assembly with aligned reads (32). Gene expression profiles were calculated for each sample and were presented as read count and normalization value which is based on transcript length and depth of coverage. The FPKM (Fragment per Kilobase of transcript per Million mapped reads) was used as a normalization value. RNA-seq data were analyzed using the R package DeSeq2 v1.26.0 for differential gene expression (33, 47). Differentially expressed genes (DEGs) were determined for each group using the cutoff values of ± 1.5 Log2 fold change and a false discovery rate (FDR) adjusted p value (also called q-value) of < 0.05. In the heatmap analysis, we utilized Euclidean distance to quantify differences between gene expression profiles. Hierarchical clustering was conducted using the complete linkage method, which calculates distances between clusters as the maximum distance between any two points from different clusters. These analyses were performed using the DESeq2 and heatmap packages in R. Additionally, we applied Z-score normalization (row scaling) to standardize gene expression data, allowing comparison across different genes. Filtered data were also used to generate pathways, networks, and functional inference using Qiagen’s Ingenuity^®^ Pathway Analysis (IPA^®^) software (Qiagen Redwood City; www.qiagen.com/ingenuity). IPA provided Z-score analyses for canonical pathways, and diseases and biofunctions based on the experimentally observed cause-effect relationships related to transcription, expression, activation and molecular modification.

### 2.4 Immunohistochemistry

Lungs were harvested from Wuhan-, Omicron- and MA10-infected mice after cardiac perfusion with 1X PBS and fixed in 4% Paraformaldehyde (PFA). Fixed tissues were washed with 1XPBS and transferred to 30% sucrose solution for 24 hours at 4°C. Tissues were then embedded in OCT medium on dry ice. Next, 5-μm sections were cut from the left lung collected from the infected mice. Tissue sections were stained with hematoxylin and eosin (H&E) for histopathological evaluation (abcam, Catalog# ab245880). Tissue sections were stained with CD45-Alexa Fluor^®^ 488 (cell signaling technology, Catalog# D3F8Q) or CD68-Alexa Fluor^®^ 488 (cell signaling technology, Catalog# 51644) and Anti-Actin α-Smooth Muscle-Cy3™ antibody (Sigma, Catalog# C6198) overnight at 4°C. Stained sectioned were mounted with antifade mounting medium with DAPI (30). Terminal deoxynucleotidyl transferase (dUTP) nick end labeling (TUNEL) staining was performed on lung sections using an *in-situ* cell death detection kit (Roche, Catalog# 11684795910) following the manufacturer’s instructions (21). Images were acquired with the Invitrogen™- EVOS™ M5000 Cell Imaging System and analyzed using ImageJ software.

### 2.5 Cytokine and chemokine protein measurements

Lung tissues were homogenized using a Fisherbrand™ Bead Mill 24 Homogenizer (Fisher Scientific, Catalog# 15-340-163) and 1.4 mm ceramic beads (Fisher Scientific, Catalog# 15-340-153) for 30 seconds, followed by centrifugation at 10,000 RPM for 10 mins. We analyzed the lung homogenates for cytokines and chemokines using the Milliplex Mouse Cytokine/Chemokine Magnetic Bead Panel (Millipore Sigma, Catalog# MCYTMAG70PMX25BK). The sample concentrations were calculated using the Belysa^®^ Immunoassay Curve Fitting Software (Millipore Sigma) (30).

### 2.6 Statistical analysis

Data were compared across groups using one-way ANOVA, two-way ANOVA, or Kruskal-Wallis test, followed by Dunn’s or Tukey’s multiple comparisons test for unpaired data sets. We used an unpaired Student’s t-test to compare the two groups. Statistical analysis was performed using Prism software (GraphPad, version 10, San Diego, USA). P-value <0.05 was considered significant.

## 3 Results

### 3.1 Infection of K18-hACE2 with SARS-CoV-2 VOCs

**Figure 1:**
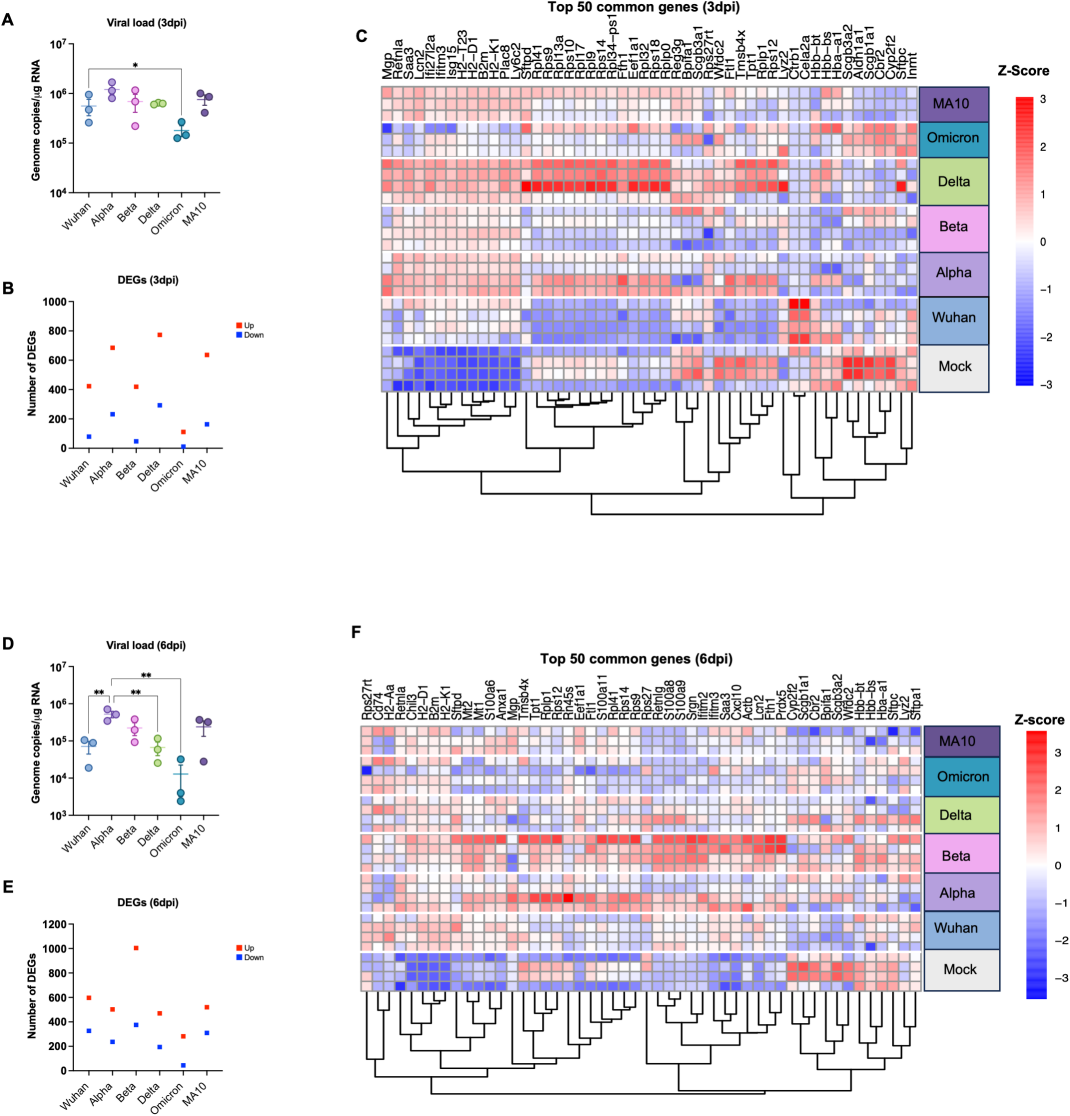
Analysis of DEGs in K18-hACE2 mice infected with SARS-CoV-2 VOCs. Six-week-old K18-hACE2 mice were intranasally infected with B.1 (Wuhan), B.1.1.7 (Alpha), B.1.351 (Beta), B.1.617.2 (Delta), B.1.1.529 (Omicron) lineages or the mouse-adapted SARS-CoV-2 (MA10). At day 3 and 6 post infection, mice were euthanized, and lung tissue was collected. **(A)** Viral RNA levels were analyzed in the lungs harvested at 3 dpi via RT-qPCR (n=3). Data are plotted for each mouse and expressed as genome copies per microgram of RNA after normalization to a standard curve. Bars indicate mean values for the group and error bars are SEM. **(B)** The number of DEGs induced in the lung at 3 dpi with Adjusted p-value (FDR) of <0.05 and |Log2 Fold change| ≥1.5; Red represents upregulated genes and blue represents downregulated genes. **(C)** Heatmap showing hierarchical clustering of the top 50 DEGs at day 3 dpi for individual samples (n=3–4 per group). Z-score normalization (row scaling) was applied to standardize the gene expression across samples. Each row represents an individual sample. **(D)** Viral RNA levels were analyzed in the lungs harvested at 6 dpi via RT-qPCR (n=3). Bars indicate mean values for the group and error bars are SEM. **(E)** The number of DEGs induced in the lung at 6 dpi. **(F)** Heatmap showing hierarchical clustering of the top 50 DEGs at day 6 dpi for individual samples (n=3–4 per group). Z-score normalization (row scaling) was applied to standardize the gene expression across samples. Each row represents an individual sample. The color represents the level of expression based of the row Z-score. Statistical significance was determined by one-way ANOVA test followed by Tukey’s multiple comparisons test or Friedman test followed by Dunn’s test. Statistical significance (*p < 0.05; **p < 0.01).

K18-hACE2 mice were infected intranasally with an infectious dose of 10^4^ PFU of the B.1 (Wuhan), B.1.1.7 (Alpha), B.1.351 (Beta), B.1.617.2 (Delta), B.1.1.529 (Omicron) variants or the mouse-adapted SARS-CoV-2 (MA10). Mice were euthanized at 3- or 6-days post-infection (dpi) and viral RNA levels in harvested lungs were evaluated by reverse transcription qPCR (RT–qPCR). At 3 dpi, we detected high levels of viral RNA in all infection groups (**Figure 1A**). At 3 dpi, viral RNA levels were comparable in Wuhan-, Beta-, Delta- and MA10- infected lungs (mean= 5.5 x10^5^, 6.8 x10^5^, 6.2 x10^5^, and 7.5 x10^5^, respectively). Alpha-infected lungs had the highest levels of viral RNA in the lungs at 3 dpi (mean= 1.2 x10^6^). In contrast, Omicron-infected lungs had the lowest levels of viral RNA in the lungs at 3 dpi (mean= 1.7 x10^5^). We also detected moderate levels of viral RNA in all infection groups at 6 dpi (**Figure 1D**). At 6 dpi, viral RNA levels in Delta-infected lungs were comparable to Wuhan-infected lungs (mean= 6.6 x10^4^ and 7.1 x10^4^, respectively). Compared to Wuhan-infected lungs, higher viral load was detected in Alpha-, Beta- and MA10-infected lungs (mean= 5.2 x10^5^, 2.2 x10^5^ and 2.4 x10^5^, respectively). In contrast, low viral load was detected in Omicron-infected lungs at 6 dpi (mean= 1.2 x10^4^).

Additionally, lung samples (three to four mice per group per time point) were subjected to RNA-seq analysis. Differentially expressed genes (DEGs) were identified by comparing the average gene expression of virus-infected lung transcriptomes with the average gene expression of mock-infected samples using the DESeq R package. An adjusted p value (FDR-value < 0.05) and fold change (FC) ratio (|Log2 (FC)| ≥1.5) were used to determine the DEGs (**Supplementary Tables 1**-**12**). Next, we determined the number of DEGs for each infection group at both time points as shown in the volcano plots (**Supplementary Figure 1**). **Tables 2** and **3** show the top ten upregulated and downregulated genes for each variant based on the absolute Log2 fold change value at days 3 and 6, respectively. Key immune markers such as Cxcl10, Zbp1, Ifit3, Isg15, Rsad2, and Irf7 were consistently upregulated across variants.

**Table 2:** Top up- and down-regulated DEGs for each variant at 3 dpi based on the absolute Log2 fold change value.

3 dpi	Wuhan	Alpha	Beta	Delta	Omicron	MA10
Up-regulated DEGs	*Cxcl10*	*Cxcl10*	*Cxcl10*	*Saa3*	*Irf7*	*Cxcl10*
	*Amy2a5*	*Saa3*	*Irf7*	*Cxcl10*	*Cxcl10*	*Saa3*
	*Ctrb1*	*Rsad2*	*Rsad2*	*Ccl2*	*Rsad2*	*Irf7*
	*Cela2a*	*Ccl2*	*Saa3*	*Cxcl9*	*Ifit3*	*Ccl2*
	*Try4*	*Irf7*	*Ly6i*	*Irf7*	*Ly6i*	*Rsad2*
	*Rsad2*	*Cxcl9*	*Isg15*	*Rsad2*	*Zbp1*	*Cxcl9*
	*Pnlip*	*Sprr1a*	*Ccl2*	*Ccl7*	*Ifit3b*	*Sprr1a*
	*Prss2*	*Ccl7*	*Zbp1*	*Isg15*	*Isg15*	*Ifi204*
	*Irf7*	*Ifi204*	*Oas3*	*Ms4a4c*	*Gm12250*	*Isg15*
	*Cela3b*	*Isg15*	*Ifi204*	*Ifi204*	*Ifit1*	*Ccl7*
Down-regulated DEGs	*Mb*	*Myh6*	*Cyp1a1*	*Cyp1a1*	*Mir6935*	*Colq*
	*Sln*	*Mb*	*Eln*	*Ccl21a*	*Hist1h1e*	*Cd209a*
	*Myl7*	*Myl7*	*Ctrb1*	*Rtkn2*	*Dusp1*	*Mir6935*
	*Myl4*	*Ckm*	*Hist1h1e*	*Fendrr*	*Cyp1a1*	*Cyp1a1*
	*Ckm*	*Cyp1a1*	*Cela2a*	*Colq*	*Spon2*	*Cyp2f2*
	*Myl3*	*Car3*	*Ces1f*	*Neat1*	*Hsph1*	*Ces1g*
	*Tnnc1*	*Sln*	*Npr3*	*Myh6*	*Fos*	*Mgl2*
	*Cox7a1*	*Tnni3*	*Ces1g*	*Carmn*	*1810011O10Rik*	*Hist1h1e*
	*Ckmt2*	*Myl4*	*Cox7a1*	*Rgs11*	*Cd209a*	*Ces1f*
	*Csrp3*	*Tff2*	*1810011O10Rik*	*Cd209a*	*Serpinh1*	*Gsta2*

**Table 3:** Top up- and down-regulated DEGs for each variant at 6 dpi based on the absolute Log2 fold change value.

6 dpi	Wuhan	Alpha	Beta	Delta	Omicron	MA10
Up-regulated DEGs	*Cxcl10*	*Cxcl10*	*Cxcl2*	*Cxcl10*	*Cxcl10*	*Cxcl10*
	*Cxcl9*	*Irf7*	*Il1r2*	*Cxcl9*	*Cxcl9*	*Cxcl9*
	*Irf7*	*Arg1*	*Acod1*	*Irf7*	*Irf7*	*Irf7*
	*Saa3*	*Ccl2*	*Cxcl10*	*Ubd*	*Ly6i*	*Trem2*
	*Gm12250*	*Cxcl9*	*Il1b*	*Il1r2*	*Saa3*	*Sprr1a*
	*Iigp1*	*Saa3*	*Cxcl3*	*Gm12250*	*Gzmb*	*Isg15*
	*Tgtp1*	*Rsad2*	*Saa3*	*Tgtp1*	*Gm12250*	*Rsad2*
	*Serpina3g*	*Trem2*	*Gm5483*	*Iigp1*	*Ubd*	*Saa3*
	*Ubd*	*Sprr1a*	*Retnlg*	*Saa3*	*Il18bp*	*Ifi204*
	*Serpina3f*	*Il1r2*	*Stfa2l1*	*Igtp*	*Zbp1*	*Gpnmb*
Down-regulated DEGs	*Myl7*	*Hbb-bt*	*Spon2*	*Cyp1a1*	*Cyp1a1*	*Hbb-bt*
	*Myl4*	*Mb*	*Cyp1a1*	*Apln*	*Ctrb1*	*Mb*
	*Apln*	*Car3*	*Aplnr*	*Ccl21a*	*Hist1h1e*	*Ptgds*
	*Cyp1a1*	*Ckm*	*B430010I23Rik*	*Aplnr*	*Mir6935*	*Ckm*
	*Mybphl*	*Cfd*	*Apln*	*Cd209a*	*Cela2a*	*Colq*
	*Myh6*	*Myl4*	*Sox18*	*B430010I23Rik*	*Fabp1*	*Actc1*
	*Car3*	*Myl7*	*Ushbp1*	*Colq*	*Cd209a*	*Apln*
	*Cyp2a5*	*Ccl21a*	*Colq*	*Spon2*	*Apln*	*Ces1f*
	*Ces1f*	*Actc1*	*Pcdh12*	*Fabp1*	*Aplnr*	*Mir6935*
	*Tff2*	*Apln*	*Glp1r*	*Hc*	*Ces1g*	*Aplnr*

Infection with the Delta variant induced the highest number of significantly up- and down-regulated genes at 3 dpi (**Figure 1B**). In contrast, infection with the Omicron variant induced the lowest number of DEGs at 3 dpi (**Figure 1B**). We next applied hierarchical clustering utilizing Euclidean distance and the complete linkage approach to measure gene expression dissimilarities based on Z-score normalization (row scaling). **Figure 1C** presents the top 50 common genes identified by highest expression variance across samples, calculated from row variance of normalized counts. Hierarchical clustering analysis identified four distinct clusters, each with clear biological implications (**Figure 1C**). Cluster 1, characterized by enrichment of genes such as *Retnla*, *Ifit27l2a, Saa3, Plac8, Ly6c2, B2m, H2-T23, H2-D1*, and *H2-K1*, distinguished the variants into two groups. One group, consisting of Delta, Beta, Alpha, and MA10 variants, exhibited upregulation of these genes, while the other group, comprising Wuhan and Omicron variants, did not show such upregulation. Considering the role of cluster 1 genes in antigen presentation and host immune responses, these findings align with viral data indicating that Wuhan and Omicron variants replicate less efficiently and consequently induce a modest immune response compared to other variants, including Delta, Beta, Alpha, and MA10 (**Figure 1A**). The second cluster, defined by enrichment of ribosomal protein (Rps) genes including *Rpl41, Rps9, Rps10, Rpl9, Rpl17, Rpl32*, and *Rps18*, further separated the variants into two distinct groups. Lung samples infected with Delta and most Alpha samples exhibited upregulation of these genes, while samples infected with other variants did not show any increase. Interestingly, the cluster 3 genes such as *Ftl1, Tpt1*, and *Rps12* exhibited downregulation across all variants except Delta and Alpha. Conversely, most genes within the cluster 4 (*Inmt, Cbr2*, and *Aldn1a1*) demonstrated downregulation across all variants except in Omicron- and Wuhan-infected samples. Interestingly, *Ctrb1* and *Cela2a* genes were only upregulated in Wuhan-infected mice. It is noteworthy that highly upregulated genes across the variants such as Cxcl10, Rsad2, and Irf7 (**Table 2**) are absent from the 3 dpi heatmap in **Figure 1C**. **Table 2** lists the top 10 DEGs based on absolute log₂ fold change from DESeq2 analysis, highlighting genes with the most pronounced expression changes. In contrast, **Figure 1C** presents the top genes exhibiting the highest expression variance across all samples. Thus, although Cxcl10, Rsad2, and Irf7 showed substantial fold changes, their expression variability was insufficient for inclusion among the most variable genes at 3 dpi.

At 6 dpi, the Beta variant induced the highest number of significantly up- and down- regulated genes (**Figure 1E**). In contrast, infection with the Omicron variant induced the lowest number of DEGs at 6 dpi (**Figure 1E**). Hierarchical clustering analysis of gene expression profiles identified four distinct clusters (**Figure 1F**). Cluster 1, characterized by enrichment of genes including *Rps27rt*, *H2-k1*, and *H2-D1* demonstrated upregulation after infection with all the variants compared to the mock group. Cluster 2, defined by enrichment of ribosomal protein (Rps) genes separated the variants into two distinct groups. Most lung samples infected with Beta and Alpha variants exhibited upregulation of these genes, while samples infected with other variants did not show any increase. Similarly, in the cluster 3, upregulation of key immune genes such as *Cxcl10*, *Ifitm2*, and *S100a8* was only observed in lung samples infected with Beta, Alpha and Wuhan variants. Conversely, most genes within the cluster 4 demonstrated downregulation across all variants except Beta and Alpha. Overall, samples infected with the Beta and Alpha variants exhibited a robust host response, corresponding with the elevated viral titers detected in these animals at 6 dpi (**Figure 1D** **).**

### 3.2 Common and variant-specific DEGs following infection with SARS-CoV-2 VOCs

**Figure 2:**
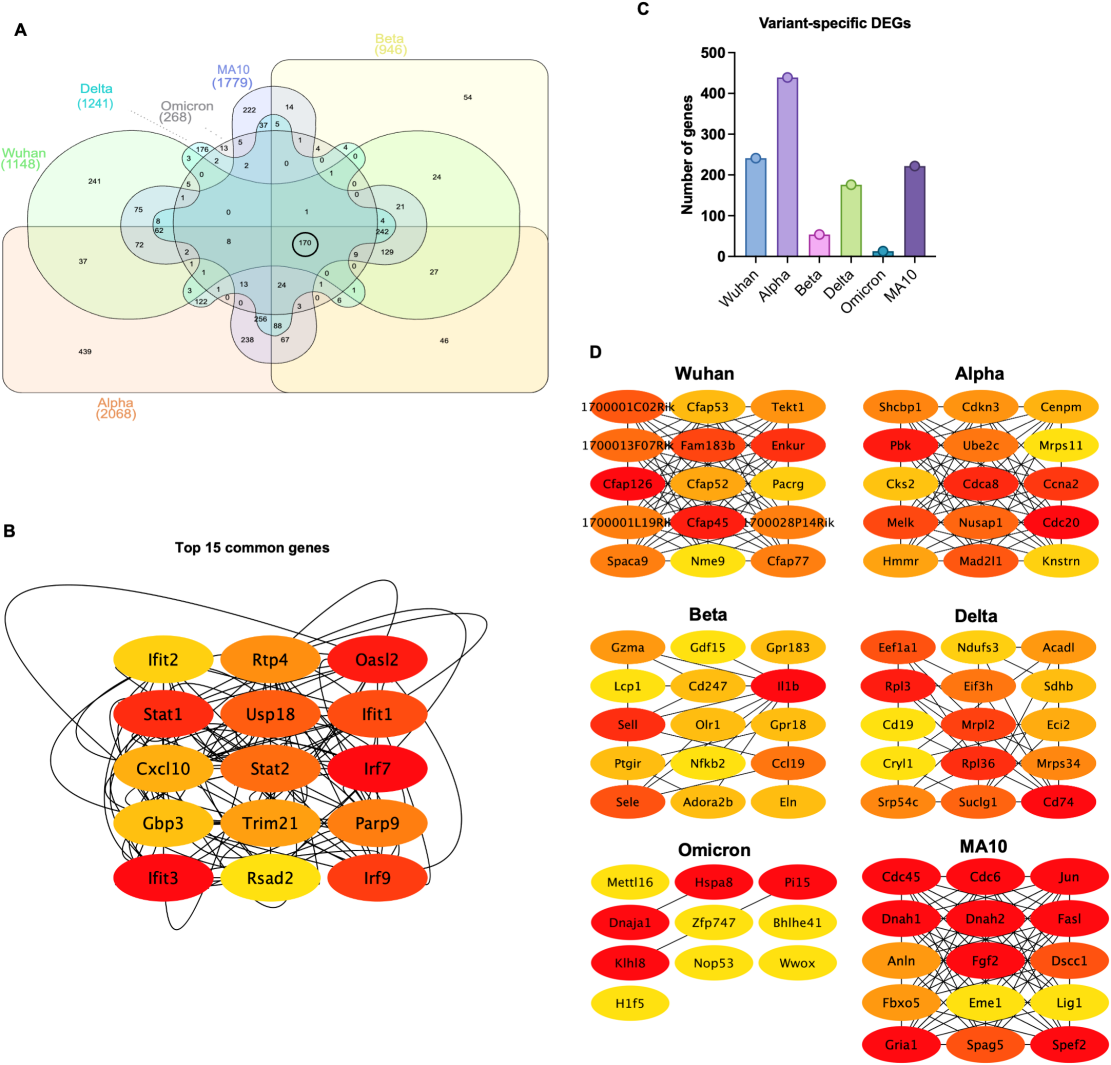
Common and variant-specific DEGs induced in the lungs following infection with SARS-CoV-2 VOCs. **(A)** Venn diagram showing the overlap of the DEGs among variants. **(B)** Network visualization of the top 15 hub genes identified from the overlapping DEGs using the CytoHubba plugin, ranked based on the MCC (Maximal Clique Centrality) algorithm, where red indicates the highest-ranking genes and yellow represents lower-ranking genes. **(C)** Bar chart displaying the number of variant-specific DEGs unique to each SARS-CoV-2 variant. **(D)** Network visualization of key variant-specific hub genes identified by CytoHubba and ranked using the MCC algorithm. Genes with higher ranks are indicated in red, while lower-ranking genes are shown in yellow, highlighting essential genes associated with variant-specific responses.

Persistent DEGs genes (common between 3 and 6 dpi) for each variant were used to generate Venn diagram to identify common genes among variants and variant-specific genes (**Figure 2A**). Comparison of DEGs revealed 170 common genes among all variants at both 3 and 6 dpi (**Table 4**). These common genes included key immune factors such as *Cxcl10, Zbp1, Ifit3, Isg15, Rsad2*, and *Irf7*.

**Table 4:** Common genes shared across variants.

Common genes						
*Ifit3*	*Cd209a*	*Oas1c*	*Mcemp1*	*Ifi27l2b*	*Batf2*	*Oasl2*
*Il18bp*	*Hist1h1e*	*Trim25*	*H2-M3*	*Psmb9*	*H2-Q8*	*Trex1*
*Oas1a*	*1810011O10Rik*	*Tbrg1*	*Irf9*	*Stat2*	*Clec7a*	*Gbp10*
*Oas1g*	*Wfdc1*	*Bhlhe40*	*Trim21*	*Tyrobp*	*H2-Q6*	*Tgtp2*
*Apol9a*	*Tril*	*Tor1aip1*	*Sesn2*	*H2-Q4*	*Gm6904*	*Phf11d*
*Gm12250*	*Hepacam2*	*Rnf114*	*H2-L*	*Slc39a2*	*H2-T22*	*Sirpb1a*
*Apod*	*Adamts17*	*Shisa5*	*H2-T-ps*	*Sp140*	*H2-T23*	*Usp18*
*Ly6c2*	*Serf1*	*Samhd1*	*Trafd1*	*Ear3*	*Slfn2*	*LOC100038947*
*Oas3*	*Prdm8*	*Tap2*	*Dusp5*	*Bst2*	*Rtp4*	*Gbp5*
*Mx1*	*Sh3bgr*	*H2-Q2*	*Ly6a*	*Rbm3*	*B430306N03Rik*	*Igtp*
*Oasl1*	*Hsph1*	*Nuak2*	*Slc7a5*	*Stat1*	*Tap1*	*Irgm1*
*Ly6i*	*Hist2h3c2*	*Chaf1b*	*Rasa4*	*Trim30d*	*Lcn2*	*Iigp1*
*Zbp1*	*Angpt4*	*Psme1*	*Ms4a8a*	*Lgals9*	*Ctss*	*Ifi27l2a*
*Isg15*	*Clec11a*	*Ogfr*	*Parp9*	*Pld4*	*Gm4951*	*Cd274*
*Ifi204*	*Cd207*	*Gm15133*	*Xdh*	*Ube2l6*	*Gbp3*	*H2-Q9*
*Cxcl9*	*Hist2h2be*	*Ly6e*	*H2-Q1*	*Cybb*	*Gpnmb*	*Ifit2*
*Irf7*	*Mest*	*Coro2a*	*Pim1*	*H2-Q5*	*H2-T9*	*Dhx58*
*Rsad2*	*Hspa4l*	*Cdt1*	*H2-D1*	*Irgm2*	*Lgals3bp*	*Fcgr4*
*Saa3*	*Proca1*	*Rab20*	*Parp12*	*Psmb8*	*Siglec1*	*Apol9b*
*Cxcl10*	*Fkbp4*	*9930111J21Rik2*	*H2-T10*	*Epsti1*	*Sirpb1b*	*Tgtp1*
*Ifit1*	*C430049B03Rik*	*Parp10*	*Tnf*	*H2-Q7*	*Ifi47*	
*Ms4a6c*	*Ahsa2*	*Nfkbie*	*Dtx3l*	*Cd68*	*Gm5431*	
*Slfn1*	*Nat8f7*	*Laptm5*	*Spi1*	*Ddx60*	*Gbp6*	
*Cfb*	*Hsp90ab1*	*Zc3h12a*	*B2m*	*Xaf1*	*AI607873*	
*Ifit3b*	*Ptgs1*	*Ear12*	*Olfr56*	*Mpeg1*	*Cyp4f18*	

Next, shared genes were subjected to network analysis and visualized using Cytoscape. The top 15 hub genes common among all variants were identified using the Maximal Clique Centrality (MCC) algorithm and CytoHubba plugin. Most of these genes were involved in the interferon response such as regulatory factor (*Irf*)-*7 and -9*, *Stat1*, *Stat2*, *Ifit1, Ifit2*, *Ifit3, Usp18, Trim21, PARP9, and Rsad2.* Other genes known for their role in viral restriction such as *Rtp4* and *Gbp3* were also uniformly shared among variants. Furthermore, the chemokine *Cxcl10* was also shared among all variants (**Figure 2B**).

Next, we identified variant-specific genes (**Supplementary Table 13**). The Beta and the Omicron variants had the lowest number of unique genes (**Figure 2C**). Unique variant-specific genes were subjected to network analysis and visualized using Cytoscape. The top hub genes for each variant were identified using the MCC algorithm and CytoHubba plugin (**Figure 2D**). In Wuhan-infected lungs, we detected the dysregulation of genes involved in cilium-dependent cell motility processes including genes in the cilia- and flagella-associated protein family (*Cfap*) and in the tektin (*Tekt*) family. For example, the dysregulated genes included *Cfap45*, *Cfap52*, *Cfap53*, *Cfap77*, and *Cfap126*. In Alpha-infected lungs, cell cycle-related genes such as cell division cycle 20 (*Cdc20*), Cyclin-Dependent Kinase Subunit 2 (*Cks2*), Cell Division Cycle Associated 8 (*Cdca8*), Cyclin A2 (*Ccna2*), and Cyclin-Dependent Kinase Inhibitor 3 (*Cdkn3*) were dysregulated. In Beta-infected lungs, dysregulation of G protein-coupled receptor genes such as *Gpr18* and *Gpr183* was observed. In Delta-infected lungs, we found significant changes in the genes associated with immune cell activation and ribosomal protein family such as the *Cd19*, *Cd74*, *Rpl3*, and *Rpl36*. In Omicron-infected lungs, genes associated with protein folding and degradation including the Heat Shock Protein Family A Member 8 (*Hspa8*), Peptidase Inhibitor 15 (*Pi15*) and the Kelch-like protein 8 (*Klhl8*), and with transcriptional regulation, such as the Zinc Finger Protein 747 (*Zfp747*), Basic Helix-Loop-Helix Family Member E41 (*Bhlhe41*), and Methyltransferase-like 16 (*Mettl16*) were dysregulated. In MA10-infected lungs, unique genes were *Cdc6*, *Cdc45*, dynein heavy chain 1 and 2 (*Dnah1* and *Dnah2*).

### 3.3 Enriched pathways following SARS-CoV-2 VOCs infection

**Figure 3:**
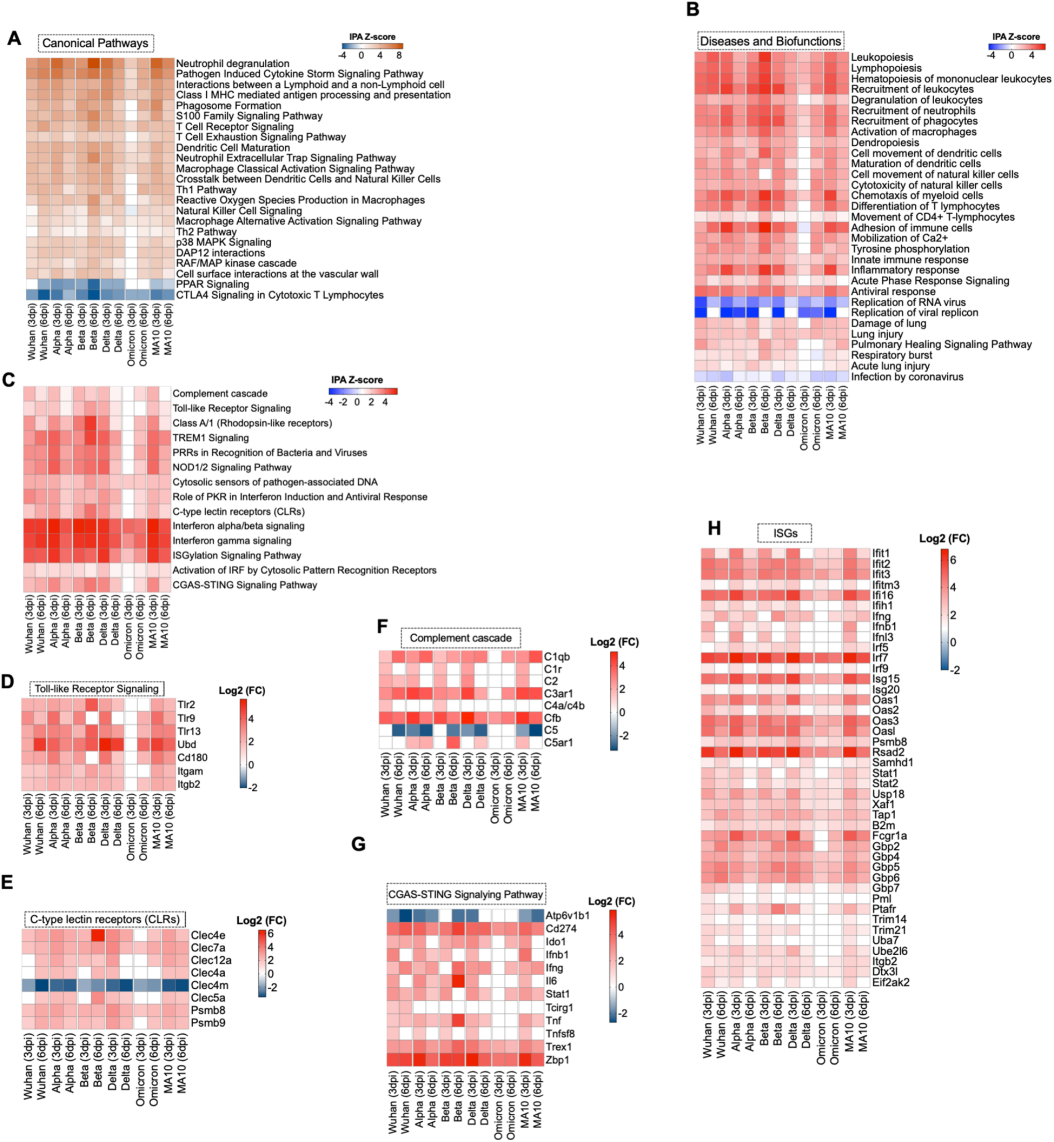
Functional enrichment analysis of DEGs. **(A)** Comparative analysis of the top canonical pathways induced in the lungs by SARS-CoV-2 generated by Ingenuity pathway analysis (IPA). **(B)** Comparative analysis of diseases and biofunctions in the lungs in response to SARS-CoV-2 infection at days 3 and 6 post infection. **(C)** Enrichment analysis of DEGs with IPA. The expression levels of genes associated with **(D)** Toll-like receptor, **(E)** C-type lectin receptors, **(F)** complement system, **(G)** cGAS-STING pathway and (**H**) Interferon-stimulated genes. The expression levels are relative to the mock-infected K18-hACE2 lung samples and are expressed in Log2 fold change.

Ingenuity Pathway Analysis (IPA) software was used to interrogate the top canonical pathways induced in the lungs after each infection over time based on the activation-z score value. Pathways such as neutrophil degranulation and pathogen induced cytokine storm were among the top activated canonical pathways. At 3 dpi, Alpha-, Delta-, and MA10-infected lungs showed higher activation of these pathways compared to Wuhan-infected lungs. However, at 6 dpi the most activation was observed in Beta-infected lungs. At both 3 and 6 dpi, Omicron-infected lungs showed the lowest activation of canonical pathways compared to other variants. In addition, pathways associated with macrophage activation such as production of nitric oxide and reactive oxygen species and macrophage classical activation pathway were activated in all variants. Pathways associated with immune system activation such as dendritic cell maturation, T cell receptor signaling and natural killer cell signaling were among the activated canonical pathways at both 3 and 6 dpi. Notably, despite the lower viral load in the lungs by 6 dpi, pathways associated with the acute-phase response of inflammation were still activated in all variants. In contrast, CTLA4 signaling in cytotoxic T lymphocytes was inhibited in all variants. All variants showed inhibition of PPAR signaling pathways except Omicron-infected lungs (**Figure 3A**). We also examined the enriched diseases and biofunctions. Biofunctions associated with an acutely activated immune system marked by several types of immune cells proliferation, recruitment, infiltration, adhesion, and activation, showed significant activation in all variants, albeit at lower levels in Omicron-infected lungs especially at 3 dpi. Processes such as inflammatory response and antiviral response also showed significant activation in all variants. We also observed the inhibition of replication of viral replicon pathway at 3 dpi in all variants. Interestingly, inhibition of this pathway was still observed in Alpha- and Omicron-infected lungs at 6 dpi. Despite the observed inhibition of replication of viral replicon pathway, the viral load in Alpha-infected lungs remained high at 6 dpi. On the other hand, the viral load in Omicron-infected lungs was low at 6 dpi (**Figure 3B**). Further, we observed the low activation of cell movement and immune infiltration pathways in Omicron-infected lungs at 3 dpi. Genes associated with transmigration of leukocytes are shown in **Supplementary Figure 2A**. To further validate our observation, we deployed CD45^+^ labeling of Wuhan- and Omicron-infected lungs harvested at 3 dpi. We detected lower CD45^+^ infiltrates in the lungs of Omicron-infected mice compared to Wuhan-infected lungs (**Supplementary Figure 2B**).

### 3.4 Activation of pathogen recognition pathways following SARS-CoV-2 infection

Infection with Wuhan, Alpha, Beta, Delta or MA10 resulted in the activation of the host pattern recognition receptors such as Toll-Like Receptors (TLRs), C-type Lectin Receptors (CLRs), and NOD1/2 receptors as early as 3 dpi. However, activation of CLRs and NOD1/2 receptors was induced at 6 dpi in Omicron-infected lungs. No activation of TLRs was detected in Omicron-infected lungs at 3 or 6 dpi. Activation of IRF by Cytosolic Pattern Recognition Receptors was observed at 3 and 6 dpi in all groups (**Figure 3C**). We further delineated the genes associated with the activated pathways. We found significant upregulation in *Tlr2*, *Tlr9*, and *Tlr13* (**Figure 3D**). C-type lectin receptors (CLRs) are PRRs that interact with pathogens via carbohydrate structures, resulting in enhanced antigen presentation (34). We found a significant upregulation of *Clec7a*, also known as Dectin-1, in all variants at 3 and 6 dpi. In contrast, the *Clec4m* gene that encodes for the L-SIGN receptor was significantly downregulated in all variants at 3 and 6 dpi (**Figure 3E**). L-SIGN was identified as an alternative receptor for SARS-CoV (35). Recently, it was shown that L-SIGN interacted with high-mannose-type N-glycans on the SARS-CoV-2 spike protein, indicating that L-SIGN may serve as an additional receptor for SARS-CoV-2 infection (36). Furthermore, several studies identified L-SIGN as an attachment receptor that enhances ACE2-dependent infection and promotes SARS-CoV-2 trans-infection (4). The utilization of the L-SIGN during viral attachment can lead to its downregulation as a protective mechanism to reduce viral entry into host cells and limit further viral replication. Similar observations are reported for the ACE2 receptor (4).

Several lines of evidence implicated the hyperactivation of the complement system as a key component in the pathogenesis of COVID-19 (37). Our data showed the significant upregulation of *Cfb* gene in all variants at 3 and 6 dpi. Additionally, the *C1qb* and *C3ar1* genes were significantly upregulated in all infection groups except in Omicron-infected lungs at 3 dpi (**Figure 3F**).

Cyclic GMP-AMP synthase (cGAS)–stimulator of interferon genes (STING) pathway controls immunity to cytosolic double-stranded (ds)DNA, driving aberrant type I IFN responses in COVID-19 patients (38–40). The *Trex1* gene encodes for a gatekeeper enzyme of the cGAS-STING pathway to prevent aberrant activation of DNA-mediated cGAS-STING signaling. Recent studies reported that the STING agonists block SARS-CoV-2 infection via triggering the type I IFN response (41, 42). Our analysis revealed the activation of cGAS-STING pathway in SARS-CoV-2-infected lungs. However, the *Trex1* gene was also significantly upregulated in all infection groups at 3 and 6 dpi (**Figure 3G**).

### 3.5 Induction of anti-viral interferon and cytokines/chemokines responses

Our data indicated the robust IFN signaling in mouse lungs in response to SARS-CoV-2 infection in all the infection groups. Transcriptomic analysis showed the upregulation of Interferon Regulatory factors (IRFs) that are key regulators of type I IFNs expression. For example, *Irf7* showed a significant increase in gene expression at 3 and 6 dpi in all variants. We also found that interferon stimulated factor 15 (*Isg15*) exhibited significant differential expression in all variants. Additionally, interferon-induced protein with tetratricopeptide repeats 2 and 3 (*Ifit2* and *Ifit3*) and Gamma-interferon-inducible protein 16 (*Ifi16*) were significantly increased in all variants (**Figure 3H**).

SARS-CoV-2 infection also resulted in a robust increase in the expression levels of cytokines and chemokine genes. Genes associated with inflammation such as *Il1rn*, *Cxcl10*, *Cxcl9*, *Ccl2*, *Ccl4*, and *Ccr5* showed increased expression in all variants at 6 dpi. In addition, genes belonging to the tumor necrosis factor cytokine family (TNF), including *Tnfaip8l2*, *Tnfrsf1b*, and *Tnfsf9* were upregulated in the infected lungs at 6 dpi (**Supplementary Figure 3**).

### 3.6 Activated cell death pathways in the lungs following SARS-CoV-2 infection

**Figure 4:**
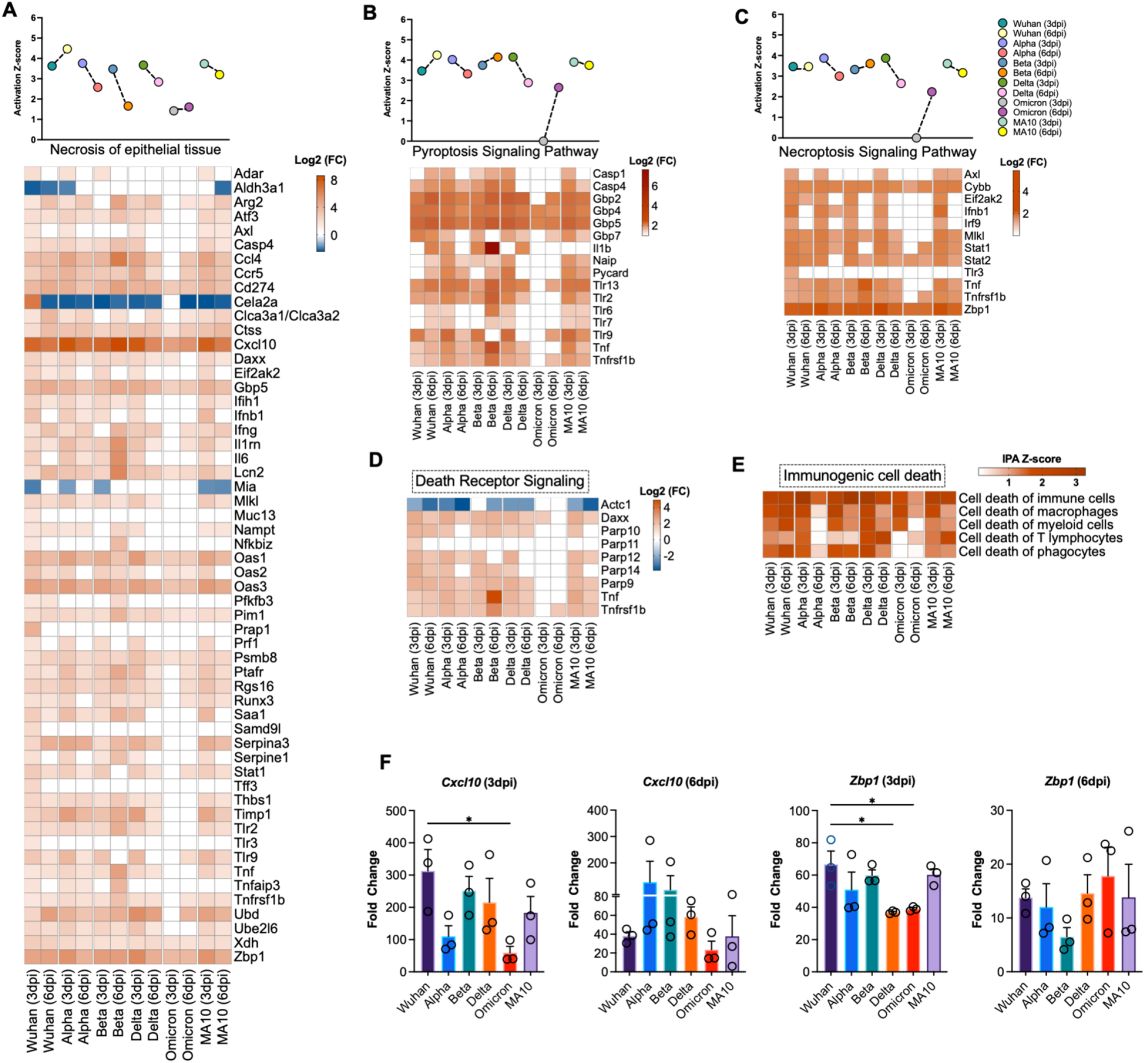
Activation of cell death pathways **in** SARS-CoV-2 infected lung tissue. Activation Z-score and DEGs of **(A)** necrosis of epithelial tissue, **(B)** pyroptosis signaling pathway and **(C)** necroptosis signaling pathway. **(D)** Differential expression of the genes associated with death receptor signaling determined by IPA. **(E)** Heatmap of activation Z-score of enriched immunogenic cell death pathways determined by IPA. The expression levels were calculated relative to the mock-infected lung samples and are expressed in Log2 fold change. **(F)** Expression level of *Cxcl10* and *Zbp1* was validated by RT-qPCR. Fold change of each gene was first normalized to the *Gapdh* gene and was calculated in comparison to corresponding fold change in control mice lungs (n = 3 per group). Bar represents the mean ± SEM. P-values were calculated by one-way ANOVA test followed by Dunnett’s multiple comparison test. (*p < 0.05).

IPA analysis of the enriched DEGs revealed the activation of several cell death pathways including necrosis of epithelial tissue in the lungs following SARS-CoV-2 infection (**Figure 4A**). Pyroptosis and necroptosis signaling pathways showed high activation in all variants at 3 and 6 dpi except in Omicron-infected lungs at 3 dpi. An array of genes that belong to Guanylate-binding proteins (GBPs) family were significantly enriched upon SARS-CoV-2 infection; these genes include *Gbp7*, *Gbp2*, *Gbp4*, and *Gbp5* (**Figure 4B**). It was recently reported that SARS-CoV-2 Z-RNA activates ZBP1-RIPK3 necroptosis pathway, resulting in an augmentation of inflammatory response (43). Consistent with these findings, we found a robust increase in expression of Z-DNA binding protein (*Zbp1*) in all variants at 3 and 6 dpi with the highest expression observed following infection with the Alpha variant at 3 dpi (Log2 (FC) = 5.869) (**Figure 4C**). Next, we analyzed genes associated with death receptor signaling pathway. We found increased expression of several genes that belong to poly (ADP-ribose) polymerase family such as *Parp9* and *Parp10* in the infected lungs. Death domain-associated gene (*Daxx*) was among the upregulated genes implicated in death receptor signaling with the highest upregulation observed at day 3 following infection with B.1 (Log2 (FC) =2.536) (**Figure 4D**). Furthermore, our analysis revealed the significant activation of immunogenic cell death in SARS-CoV-2-infected lungs (**Figure 4E** **).**

### 3.7 Validation of RNA-seq data

To validate RNA-Seq data, we performed RT-qPCR of selected genes that are differentially expressed in SARS-CoV-2-infected lungs. Glyceraldehyde 3-phosphate dehydrogenase gene (*Gapdh*) was used to normalize the relative expression of the target genes. Consistent with RNA-seq data, *Cxcl10* and *Zbp1* genes showed similar expression trend to the gene expression levels determined by RNA-seq at 3 and 6 dpi (**Figure 4F**).

### 3.8 Z-DNA-binding protein 1 restricts SARS-CoV-2 replication in the lungs

**Figure 5:**
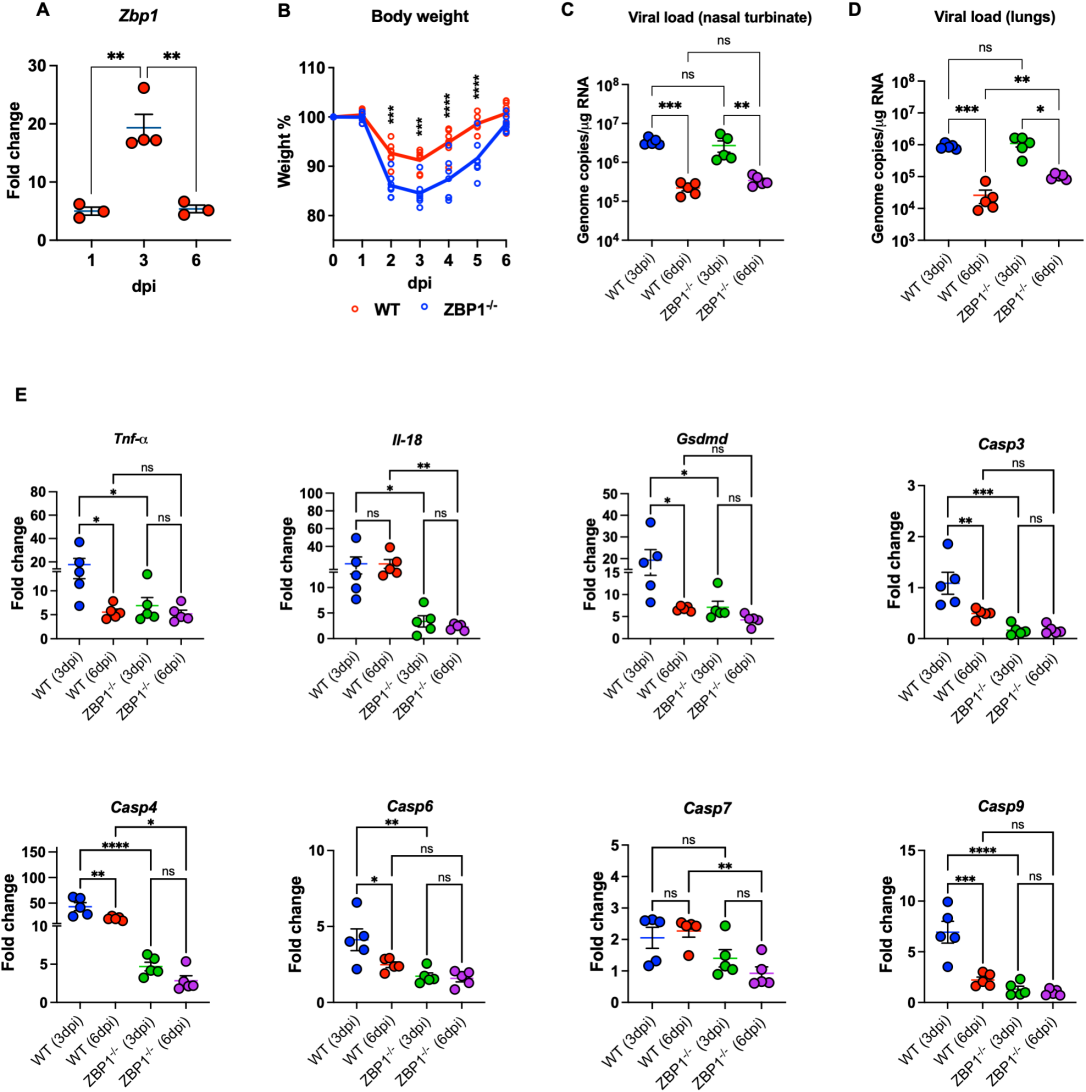
Characteristics of WT and ZBP1^-/-^ mice infected with MA10. **(A)** WT mice were infected with MA10 virus. RNA was extracted from infected lungs at days 1, 3, and 6 post infection. The expression levels of the *Zbp1* gene in the lungs were determined via RT-PCR (n=3–4 per time point). **(B)** WT and ZBP1^-/-^ mice were infected with MA10 and monitored for weight loss (n=7 per group). Significance was determined by mixed effects analysis followed by Sidak’s multiple comparisons test (*p < 0.05; **p < 0.01; ***p < 0.001, ****p < 0.0001). Viral RNA levels were analyzed **(C)** in the nasal turbinate and **(D)** in the lungs harvested at 3 and 6 dpi via RT-qPCR. Data are plotted for each mouse and expressed as genome copies per microgram of RNA after normalization to a standard curve. Bars indicate mean values for the group and error bars are SEM. Statistical significance was determined by one-way ANOVA test followed by Tukey’s multiple comparisons test. Statistical significance (*p < 0.05; **p < 0.01; ***p < 0.001. **(E)** Gene expression level of *Tnf-α*, *Il-18*, *Gsdmd*, *Casp3*, *Casp4*, *Casp6*, *Casp7*, and *Casp9* was determined in WT and ZPB1^-/-^ lungs collected at 3- and 6-days post infection. Fold change is plotted for each mouse. Fold change of each gene was first normalized to the *Gapdh* gene and was calculated in comparison to corresponding fold change in control mice lungs. Statistical significance was determined by one-way ANOVA test followed by Sidak’s multiple comparisons test. Statistical significance (*p < 0.05; **p < 0.01; ***p < 0.001, ****p < 0.0001).

We first investigated the expression levels of ZBP1 in the lungs of C57BL/6J WT mice at different time points after intranasal infection with mouse-adapted SARS-CoV-2 (MA10). We measured ZBP1 RNA levels using RT-qPCR. We detected increased levels of ZBP1 as early as day 1 post infection (mean fold change = 4.1). The expression level of ZBP1 further increased at day 3 post infection (mean fold change = 16.9) followed by a decrease at day 6 post infection (mean fold change = 4.4) (**Figure 5A**).

To determine the role of ZBP1 in SARS-CoV-2 pathogenesis, we inoculated WT and ZBP1^−/−^ mice intranasally with MA10. Interestingly, ZBP1^−/−^ mice exhibited significant weight loss compared to WT mice after challenge at days 2, 3, 4, and 5 post infection. Like WT mice, ZBP1^−/−^ mice recovered from weight loss by 6 dpi (**Figure 5B**). Next, RNA viral load was determined in the nasal turbinate and in the lungs via RT-qPCR. RNA viral load in the nasal turbinates was high at 3 dpi and significantly decreased by 6 dpi in both WT and ZBP1^−/−^ mice. No significant differences in the viral load in the nasal turbinates were detected between the groups (**Figure 5C**). In the infected lungs, RNA viral load was high at 3 dpi and significantly decreased by 6 dpi in both WT and ZBP1^−/−^ mice. No significant differences were detected between WT and ZBP1^−/−^ mice at 3 dpi. Interestingly, at 6 dpi, RNA viral load in ZBP1^−/−^ mice were significantly higher compared to WT mice (**Figure 5D**).

### 3.9 ZBP1 deficiency attenuates virus-induced cell death in the lungs following SARS-CoV-2 infection

We measured the expression levels of cell death-associated markers in infected lungs using RT-qPCR (**Figure 5E**). We first measured the levels of Tumor necrosis factor-α (*Tnf-α)* known for its role in necroptosis. Levels of *Tnf-α* were significantly lower in the lungs of ZBP1^−/−^ mice (mean fold change = 6.9) compared to WT mice (mean fold change = 17.8) at 3 dpi. Next, we examined the levels of genes involved in pyroptotic cell death such as *Casp4*, *Gsdmd*, and *Il-18*. Caspase-4 is an inflammatory caspase; it cleaves and activates its downstream mediator Gasdermin D (GSDMD). mRNA levels of *Casp4* were significantly lower in the lungs of ZBP1^−/−^ mice (mean fold change = 4.1) compared to WT mice (mean fold change = 43) at 3 dpi. Similarly, at 6 dpi, expression levels of *Casp4* were significantly lower in the lungs of ZBP1^−/−^ mice (mean fold change = 2.8) compared to WT mice (mean fold change = 20). Pyroptotic death is mediated by inflammasome assembly, which is accompanied by GSDMD cleavage and IL-18 release. Expression levels of *Gsdmd* were significantly lower in the lungs of ZBP1^−/−^ mice (mean fold change = 6.3) compared to WT mice (mean fold change = 19.2) at 3 dpi. We also evaluated the RNA levels of *Il-18* in infected lungs. Levels of *Il-18* were significantly less in the lungs of ZBP1^−/−^ (mean fold change = 3.3) mice compared to WT mice (mean fold change = 20.3) at 3 dpi. Similarly, at 6 dpi, levels of *Il-18* were significantly lower in the lungs of ZBP1^−/−^ mice (mean fold change = 2.2) compared to WT mice (mean fold change = 20.2). Further, characterization of cell death markers associated with apoptotic cell death revealed significantly decreased expression of the apoptotic initiator *Casp9* and the executioners *Casp3*, *Casp6*, and *Casp7* in the lungs of ZBP1^−/−^ mice.

**Figure 6:**
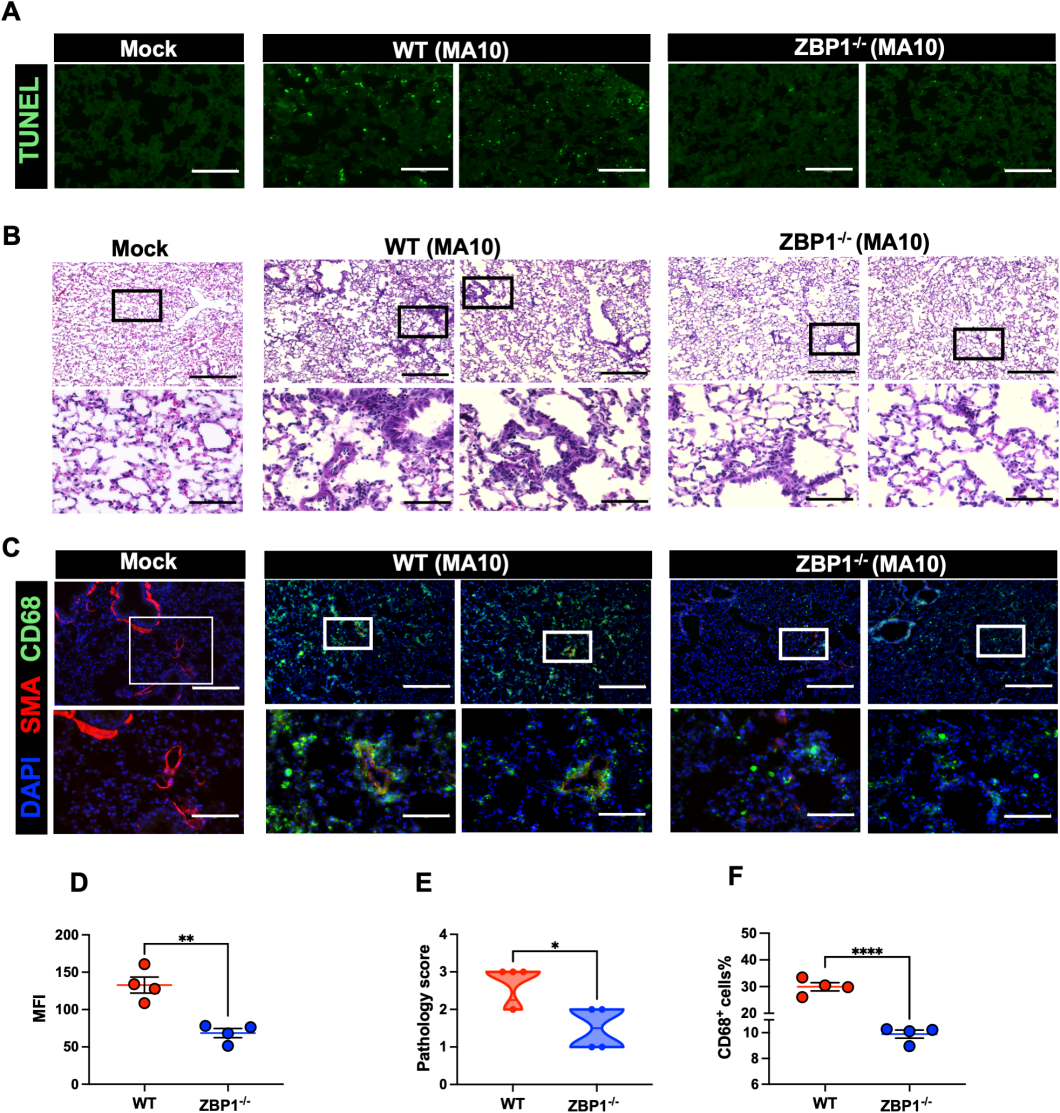
Cell death and pathology in the lungs following MA10 infection. **(A)** TUNEL assay was conducted on lung sections from mock and MA10-infected mice to detect apoptotic cells. Scale bar is 150μm. **(B)** H&E staining of mock- and MA10-infected lung tissues. Original pictures were taken with 100X magnification (scale bar 250μm). Enlarged pictures were taken with 400X magnification (scale bar 75μm). **(C)** Mock- and MA10-infected lung tissues were stained with CD58-Alexa Fluor® 488 (green), Anti-Actin α-Smooth Muscle-Cy3™ (red), and DAPI (blue). Representative images are shown. Scale bar is 250μm on original images and 75μm on enlarged images. **(D)** Mean fluorescence intensity (MFI) was determined for TUNEL-stained lung sections by ImageJ **(E)** Lung pathology score is shown for lung sections (score 1: mild infiltration and alveolar thickening, score 2: moderate infiltration and alveolar thickening, score 3: severe infiltration and alveolar thickening score, score 4: very severe infiltration and alveolar thickening) **(F)** Percentage of CD68-positive cells in MA10-infected lung sections using ImageJ. Statistical significance was determined by unpaired t test or Mann- Whitney U (*p < 0.05; **p < 0.01; ****p < 0.0001; n=4).

SARS-CoV-2-induced cell death was evaluated by direct TUNEL staining of lung tissues. Infected WT mice had increased numbers of TUNEL- positive cells in the lungs at 6 dpi. In contrast, ZBP1^−/−^ mice had low numbers of TUNEL- positive cells in the lungs (**Figure 6A**). Next, using ImageJ, we quantified the mean fluorescence intensity (MFI) in the TUNEL-stained lungs. As shown in **Figure 6D**, ZBP1^−/−^ mice had significantly lower MFI compared to WT mice. These results indicate ZBP1 play a significant role in virus-induced cell death following SARS-CoV-2 infection.

### 3.10 ZBP1 knockout results in decreased macrophage infiltration into the lungs following SARS-CoV-2 infection

Next, we investigated the impact of ZBP1 deletion on lung pathology (44, 45, 48–51). Lungs were collected from ZBP1^−/−^ and WT mice following infection with MA10. H&E staining of lung sections revealed pronounced immune cell infiltration and lung pathology in MA10-infected WT mice. However, ZBP1 depletion strongly reduced immune cell infiltration into the lungs (**Figures 6B, E**). Furthermore, depletion of ZBP1 resulted in reduced recruitment of CD68^+^ macrophages into MA10-infected lungs (**Figure 6C**). Next, using ImageJ, we quantified the percentage of CD68-positive cells in the lungs. As shown in (**Figure 6F**), ZBP1^−/−^ mice had significantly lower CD68^+^ cells in the lungs following MA10 infection. Collectively, these results indicate the role of ZBP1 in SARS-CoV-2-induced immune cell infiltration and lung pathology in mice.

### 3.11 ZBP1 deficiency attenuates the inflammatory response in the lungs following SARS-CoV-2 infection

**Figure 7:**
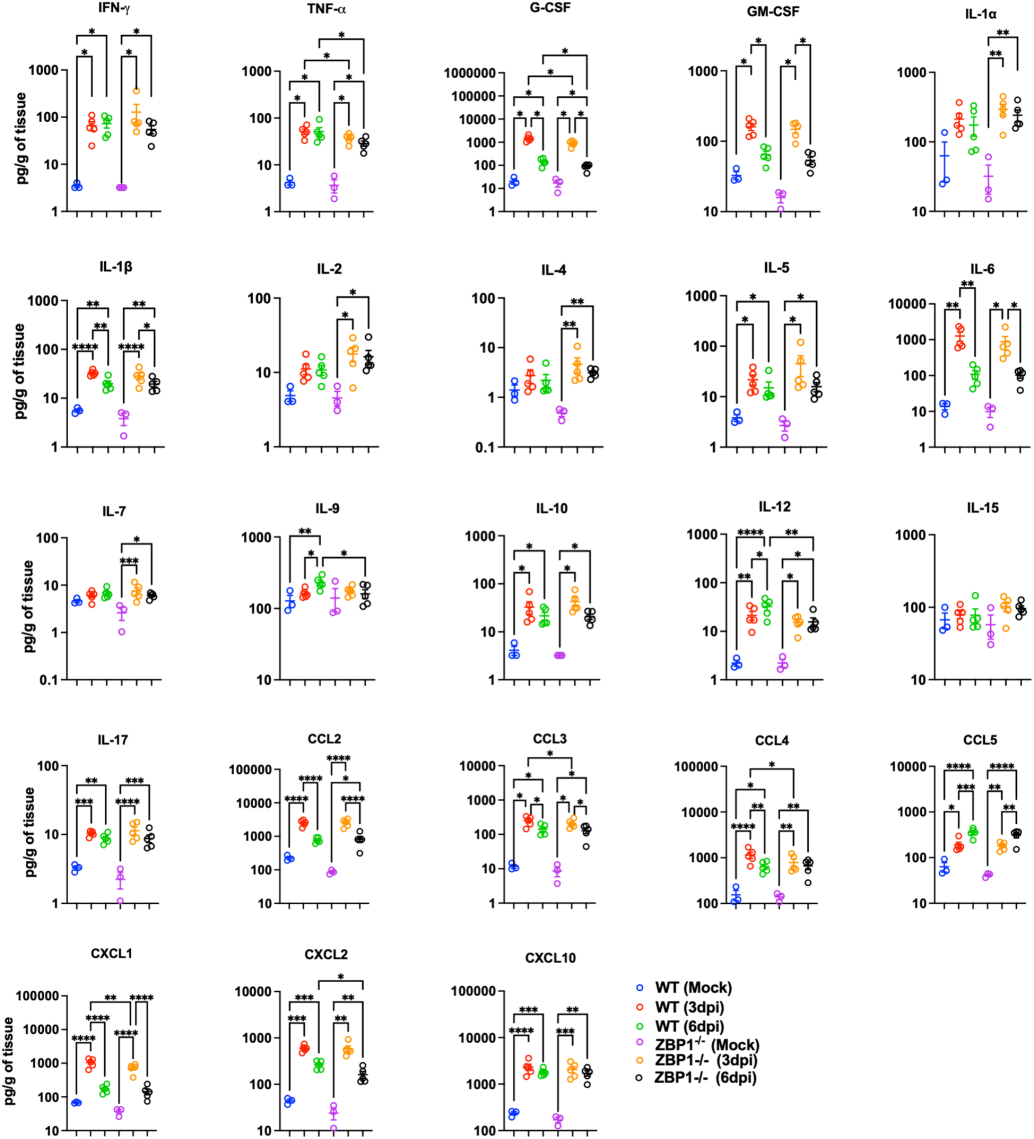
Cytokine and chemokine protein levels in the lungs of WT and ZBP1^-/-^ mice. Cytokine and chemokine protein levels in the lungs of mock- and MA10-infected animals at 3 and 6 dpi. The middle bar indicates the mean ± SEM. Each point represents an individual mouse. Statistical significance was determined by a one-way ANOVA followed by Tukey’s multiple comparisons (*p < 0.05; **p < 0.01; ***p < 0.001, ****p < 0.0001).

We also measured the protein levels of key cytokines and chemokines in lung homogenates using a multiplex immunoassay. Compared with mock samples, we detected significantly increased levels of several proinflammatory cytokines, e.g., IFN-γ, TNF-α, granulocyte colony-stimulating factor (G-CSF), granulocyte-macrophage colony-stimulating factor (GM-CSF), IL-1β, IL-5, IL-6, IL12-p70, and IL-17 in both WT and ZBP1^−/−^ infected lungs. Similarly, we detected a significant increase in C–C motif (CCL2, CCL3, CCL4, and CCL5) and C–X–C motif (CXCL1, CXCL2, and CXCL10) chemokines in both WT and ZBP1^−/−^ infected lungs. Notably, ZBP1^−/−^ mice showed significantly lower levels of TNF-α and G-CSF compared to WT mice at 3 and 6 dpi. IL-6 levels were also lower in the lungs of ZBP1^−/−^ mice; however, the difference was not statistically significant. At 3 dpi, ZBP1^−/−^ mice had significantly lower levels of CCL3, CCL4, and CXCL1 compared to WT mice. Additionally, at 6 dpi, ZBP1^−/−^ mice had significantly lower levels of IL-9, IL-12, and CXCL2 compared to WT mice. Interestingly, we detected significant increase in anti-inflammatory cytokines, IL-2 and IL-4 in only ZBP1^−/−^ infected lungs (**Figure 7**).

## 4 Discussion

In this study, we compared the transcriptional response in the lungs following infection with B.1 (Wuhan), B.1.1.7 (Alpha), B.1.351 (Beta), B.1.617.2 (Delta), B.1.1.529 (Omicron), and mouse-adapted (MA10) viruses. Our data showed that, despite the differential levels of pathogenicity and transmissibility, the transcriptional footprint of infection with the Alpha, Beta, Delta and MA10 viruses was comparable to that of the Wuhan virus. We observed the upregulation of several common host immune response-related genes in all the variants. Additionally, we identified several variant-specific genes. We also showed that Omicron variant induced lower degree of lung inflammation and pathology which is compatible with the epidemiological observations of the reduced severity of the Omicron variant in humans (44). Consistent with our observations, it was previously reported that the Omicron variant (B.1.1.529) causes attenuated disease and lower mortality rate compared to earlier variants in K18-hACE-2 mice (44). Additionally, the Beta and Delta variant showed high activation of pathways involved in the acute-phase response of inflammation and the most gene dysregulation compared to other variants. These observations are consistent with previous reports showing that these variants are more lethal and cause more severe organ lesions than the original SARS-CoV-2 WA.

Several analyses of the transcriptional response to SARS-CoV-2 compared to other respiratory viruses have been performed (48). For example, transcriptional response of SARS-CoV-2 (USA-WA1/2020) relative to other respiratory virus including SARS-CoV and MERS-CoV was previously described. It was suggested that SARS-CoV-2 induces lower levels of type I and III interferons but elevated levels of chemokines (52). Additionally, a comparative investigation of the transmissibility and antigenicity of SARS-CoV-2 VOCs has been detailed (53–55). Several studies using whole-transcriptome sequencing of autopsy lung tissue from COVID-19 patients revealed hyperinflammation, infiltration of activated macrophages but impaired T cell responses (56, 57). Also, the pathogenicity of the Alpha, the Beta, and the Delta variants was previously described (58–60). Despite the emerging data, the underlying molecular and cellular mechanisms associated with SARS-CoV-2 VOCs are not fully understood. Specifically, an in-depth comparative analysis of the host immune response following infection with these major VOCs is yet to be performed. Herein, using RNA-sequencing of the infected lungs, we demonstrated that SARS-CoV-2 induces a robust interferon response with increased expression of ISGs. Additionally, we showed that SARS-CoV-2 induces a hyperinflammatory state in the lungs characterized by increased expression of proinflammatory cytokines and chemokines. Increased infiltration of immune cells in alveolar spaces was detected in patients with ARDS (45). Consistent with these reports, IPA analysis revealed activation of immune infiltration pathways in SARS-CoV-2-infected lungs. However, our analysis revealed that unlike earlier variants, Omicron variant did not induce significant activation in transmigration of leukocytes into the lungs which was further confirmed by CD45^+^ labeling of lung tissue.

Our results also show evidence of cell death pathways activation in the lungs following SARS-CoV-2 infection. Aberrant cell death is implicated in the pathogenesis of COVID-19. However, the underlying mechanisms contributing to virus-induced cell death are still largely unclear (48). It is known that ZBP1 senses RNA viruses and initiates necroptosis in infected cells (49). It was also reported that SARS-CoV-2 infection results in the formation of Z-RNA in the cytoplasm, activating ZBP1-RIPK3 pathway. Upregulation of ZBP1 was reported in SARS-CoV-2-infected Calu-3 cells (43). In COVID-19 patients, ZBP1 expression was increased in immune cells from those who succumbed to the disease (58). In agreement with these results, our analysis revealed the significant upregulation of ZBP1 gene in infected lungs. Recently, it was suggested that ZBP1 promotes SARS-CoV-2-induced pathogenesis in infected lungs (43). In addition, ZBP1-mediated inflammatory cell death in murine macrophages and in the lungs of mice infected with SARS-CoV-2 was reported, suggesting a link between ZBP1 and pathology (58). On the other hand, several studies have reported that the loss of ZBP1 expression confers higher susceptibility to WNV or IAV infections, poor control of viral spread, and reduced survival rates in mice, suggesting the protective role of ZBP1 (28, 51). Concordantly, our data shows that ZBP1 deficiency resulted in increased SARS-CoV-2 viral load in the lungs, suggesting ZBP1 involvement in SARS-CoV-2 clearance in the lungs. Our findings are consistent with the well-established role of ZBP1 in impeding viral replication as a potent activator of innate immunity during infections (50). Importantly, we demonstrated that ZBP1 deletion resulted in reduced expression of genes involved in several cell death pathways including necroptosis, pyroptosis and apoptosis. We also showed that ZBP1 deletion significantly reduced SARS-CoV-2-induced cell death in the lungs. Further, we demonstrated that ZBP1 deletion resulted in reduced immune cell infiltration and an attenuated inflammatory response in the lungs.

Overall, we delineated the molecular and cellular changes associated with the lung injury following infection with major VOCs. We determined the common DEGs shared across the Wuhan, Alpha, Beta, Delta, Omicron, and MA10 variants as well as the key DEGs unique to each variant. We showed that SARS-CoV-2 induces a hyperinflammatory state in the lungs characterized by increased expression of proinflammatory cytokines and chemokines and aberrant infiltration of immune cells. Our data also suggested that unlike Wuhan, pre-Omicron VOCs and MA10, the Omicron variant caused attenuated disease and lower degree of lung pathology. On the other hand, the Beta and Delta variants induced the highest immune activation and severely altered gene signature. Additionally, our studies further highlighted the role of ZBP1 in alleviating SARS-CoV-2 viral burden in the lungs through the augmentation of virus-induced cell death, inflammatory response, and immune cell infiltration.

## Data Availability

The datasets presented in this study can be found in online repositories. The data presented in the study are deposited in the Sequence Read Archive (SRA) database NCBI, accession number PRJNA1238022.
