# Supplementary material for: Virus-specific host responses and gene signatures following infection with major SARS-CoV-2 variants of concern: role of ZBP1 in viral clearance and lung inflammation
Source: Front Immunol. 2025 May 9;16:1557535. doi: 10.3389/fimmu.2025.1557535 (PMC12098559; doi:10.3389/fimmu.2025.1557535)
Supplement: Supplementary file 2 [file DataSheet1.docx]

Supplementary Material


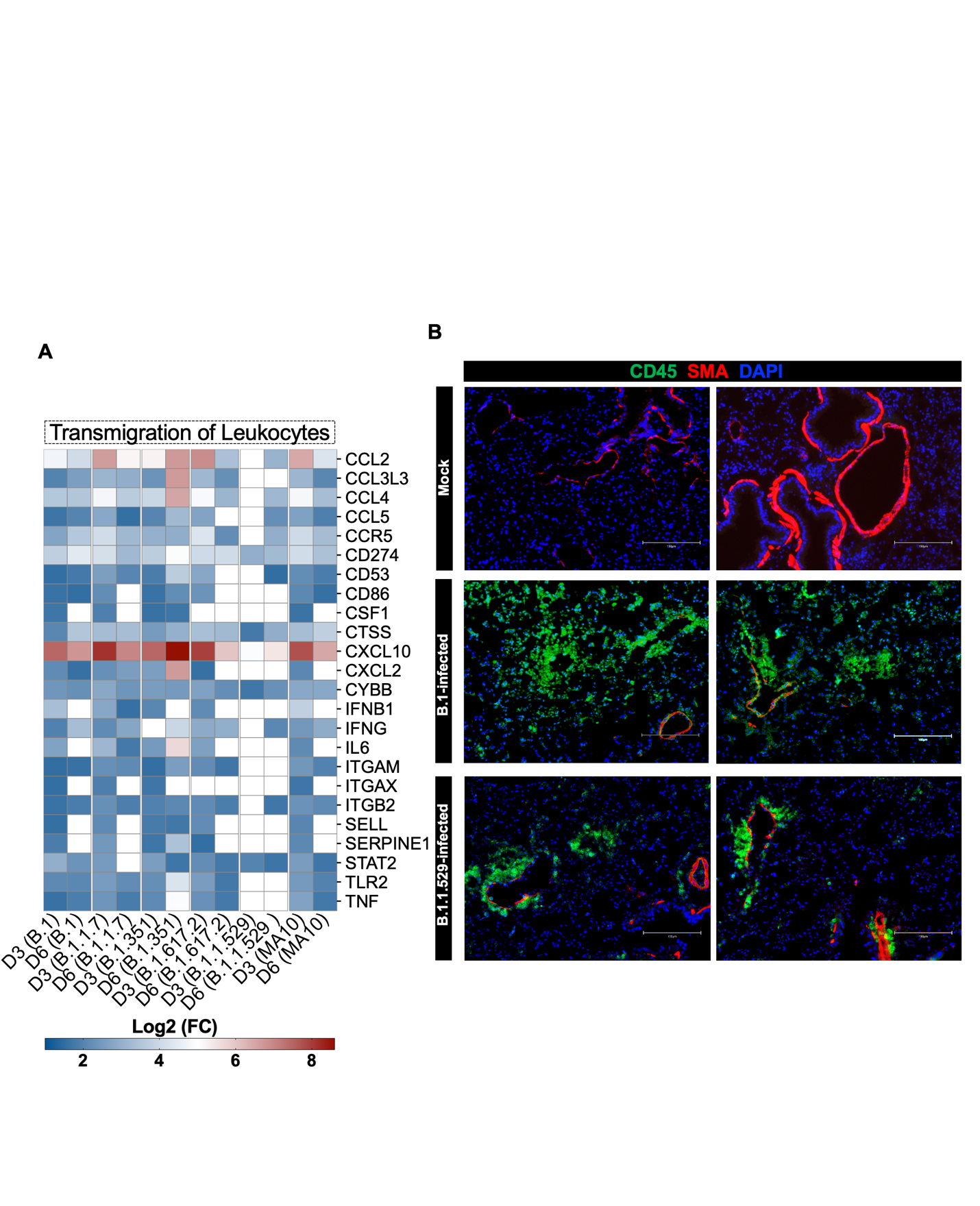


**Supplementary Figure 1: Transmigration of leukocytes in SARS-CoV-2-infected lungs. (A)** Heatmap showing the Differential expression of the genes associated with transmigration of leukocytes for all infection groups. (**B)** B.1- or B.1.1.529- infected lung tissues collected at day 3 post infection were stained with CD45-Alexa Fluor® 488 (green), Anti-Actin α-Smooth Muscle-Cy3™ (red), and DAPI (blue). Representative images are shown. Bar is 150μm.


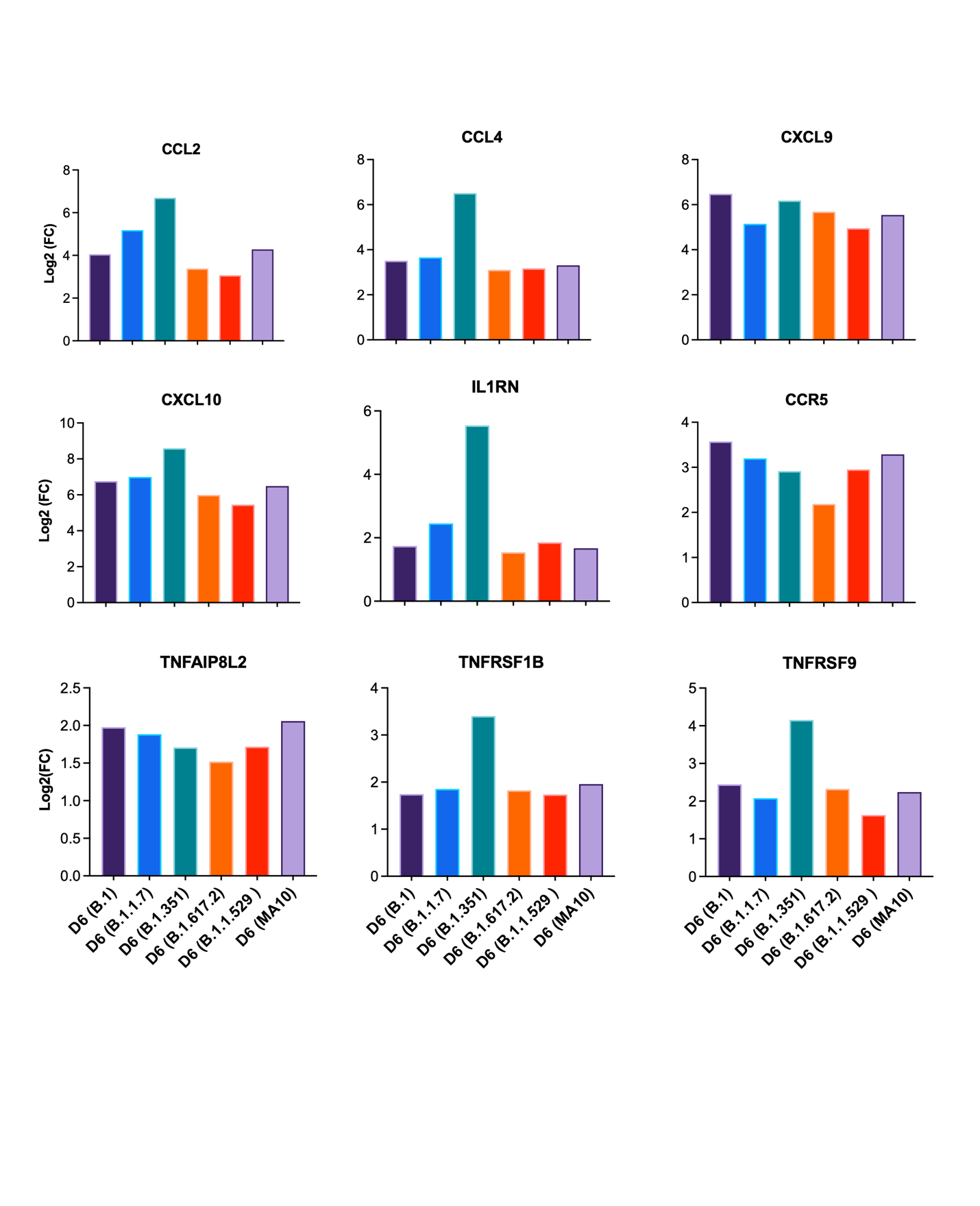


**Supplementary Figure 2. Cytokine and chemokine gene expression levels in SARS-CoV-2 infected lungs determined by RNA-Seq.** Bar graphs showing the average expression levels of CCL2, CCL4, CXCL9, CXCL10, CCR5, ILRN, TNFAIP8L2, TNFRSF9, and TNFRSF1B relative to the mock-infected K18-hACE2 lung samples (n=3-4) at day 6 post infection.
